# An updated association between TNF-α -238G/A polymorphism and gastric cancer susceptibility in East Asians

**DOI:** 10.1042/BSR20181231

**Published:** 2018-12-14

**Authors:** Hongpeng Zhao, Lixia Liu, Bo Liu, Yanmin Wang, Feng Li, Haihua Yu

**Affiliations:** 1 Department of Gastrointestinal Surgery, Shandong Provincial Qianfoshan Hospital, Shandong University, Jinan 250014, China; 2 Department of Hyperbaric Oxygen, Shandong Provincial Qianfoshan Hospital, Shandong University, Jinan 250014, China

**Keywords:** gastric cancer, meta-analysis, polymorphism, tumor necrosis factor alpha

## Abstract

Polymorphisms in the tumor necrosis factor α (*TNF-α*) gene are emerging as key determinants of gastric diseases. The TNF-α-238G/A single-nucleotide polymorphism (SNP) is the most extensively studied. However, this association is inconsistent amongst different populations. We therefore conducted an updated meta-analysis to obtain a more precise estimate of the association of TNF-α-238G/A polymorphism with gastric cancer (GC) risk. A comprehensive search of PubMed, Embase, Chinese (CNKI and WanFang) databases was performed to identify relevant studies through 5 May 2018. Odds ratio (OR) and 95% confidence interval (CI) were used to assess the strength of the association. Fourteen studies were included in our meta-analysis involving 2999 cases and 4685 controls. There was no significant association between TNF-α-238G/A polymorphism and GC risk in the overall populations. In the subgroup analysis, we found that TNF-α-238G/A polymorphism was associated with the increased risk of GC amongst Asians, especially in Chinese, but not in Caucasians. Subgroup analysis by genotyping methods revealed increased risk for other methods. In conclusion, our present meta-analysis shows that TNF-α-238G/A polymorphism is associated with the risk of GC in East Asian individuals.

## Introduction

Gastric cancer (GC) is the fourth most common malignancy worldwide [1], with a frequency that varies greatly across different geographic locations [2]. The major etiological risk factor for GC is *Helicobacter pylori* (*H. pylori*), which progresses through a multi-step process, developing from gastritis to gastric atrophy, intestinal metaplasia, dysplasia, and finally to carcinoma [3]. However, a high prevalence of *H. pylori* infection does not always result in a high incidence of GC. Only 1–2% amongst those infected will develop stomach cancer [4], suggesting that other susceptible factors, such as genetic variants or environmental differences, may be involved in gastric tumorigenesis.

Polymorphisms of inflammation-related genes have provided evidence that host genetic factors play a pivotal role in the pathogenesis of GC [5,6]. TNF-α is a potent immunomodulator and pro-inflammatory cytokine that mediates diverse pathological processes [7,8]. Several single-nucleotide polymorphisms (SNPs) have been identified in the *TNF-α* gene, mainly in the 5′-promoter regions. Several SNPs (TNF-α -857, TNF-α -1031, TNF-α -863, TNF-α -308, and TNF-α -238) in *TNF* gene have been widely reported, in which the positions -308 and -238 have been most frequently evaluated for association with GC [9–11].

The association between GC and TNF-α-308 G/A polymorphism has been confined by two meta-analyses [12,13]. This polymorphism may contribute to susceptibility of GC in Caucasians, but not in Asians. In addition, there have been three meta-analyses reporting the TNF-α-238 G/A polymorphism and GC risk [14–16]. Yu et al. [16] found TNF-α-238 G/A polymorphism is significantly associated with increased risk of GC, especially in Asians. Nevertheless, there were several limitations in this study, including duplicated studies (e.g. Wu et al. (2003); Xing et al. (2006) [17,18]), two degrees’ articles, and another study were omitted (Zang et al. (2009); Whiteman et al. (2010); Li et al. (2012) [19–21]). Furthermore, the Hardy–Weinberg equilibrium (HWE) of the control in one study was not consistent with the standard (Kamangar et al. (2006) [22]). In addition, Xu et al. [15] conducted a meta similar to Yu et al. [16] and reported an intensified risk of GC risk amongst Asians, but decreased risk of GC amongst Caucasians. Limitations within these analyses also existed, as some studies not according to HWE were included (e.g., Whiteman et al. (2010); Wu et al. (2004) [21,23]). Yin et al. [24], in fact, included two case–control studies, which was not suitable to be combined. However, Rokkas et al. [14] found no association between TNF-α -238 G/A polymorphism and GC susceptibility.

Additionally, Cen and Wu [25] and Wang et al. [26] both showed that TNF-α -857 C/T polymorphism is significantly associated with increased risk of GC. However, TNF-α -1031 and TNF-α -863 polymorphisms have neither the meta-analysis, they were just reported in signal case–control: Hamajima et al. [27] found -1031CC was not only related with reduced items, such as sex, age, but also low seropositivity. Yang et al. [28] suggested TNF-α -1031 and TNF-α -863 were associated with a significantly higher risk for GC only amongst smokers.

In summary, only the -238G/A polymorphism exists with conflicting results, although some published meta. In addition, considering the important role of TNF-α in gastric carcinogenesis and some limitations in the previous two meta-analyses, we performed an updated analysis on all eligible case–control studies to estimate the GC risk associated with -238 G/A polymorphism (including race, source of control, and genotype methods). To our knowledge, this is the most updated meta-analysis conducted to date with respect to the association between TNF-α-238 G/A polymorphism and GC risk.

## Materials and methods

### Identification of eligible studies and search criterion

A literature search of the PubMed, Embase, Web of Science, Google Scholar, WanFang, and CNKI database (updated on 11 June 2018) was conducted using combinations of the following keywords ‘polymorphism’ or ‘variant’ or ’mutation’, ‘gastric’ or ‘stomach’, ‘cancer’ or ‘carcinoma’ and ‘TNF’ or ‘tumor necrosis factor alpha’. There was no language restriction. All studies that evaluated the associations between polymorphisms of *TNF-α* gene and GC risk were retrieved. Studies that were included in our meta-analysis had to meet all of the following criteria: (i) evaluation of TNF-α gene -238 G/A polymorphism and GC risk; (ii) case–control design; (iii) availability of genotype frequency; (iv) availability of full text; and (v) genotype distributions of control consistent with HWE. Meanwhile, the following exclusion criteria were also used: (i) no control population, (ii) no available genotype frequency, (iii) HWE of controls were <0.05, and (iv) studies have not been published; for studies with overlapping or repeating data, the most recent or complete studies with the largest number of cases and controls were included and others were excluded.

### Data extraction

The following data were collected from each study: first author’s last name, year of publication, race of origin, sample size (cases/controls), each frequency number of genotype both case and control samples, study design (hospital-based (HB) and population-based (PB)), HWE of controls and genotype method.

### Statistical analysis

Risk ratios (ORs) with 95% confidence intervals (CIs) were used to measure the strength of the association between TNF-α gene -238 G/A and GC based on the genotype frequencies in cases and controls. We analyzed this relationship between TNF-α gene -238 G/A polymorphism and GC risk using three different genetic models: allelic contrast (A compared with G), heterozygote comparison (AG compared with GG), and dominant genetic model (AA+AG compared with GG). Different ethnic descents were categorized as Caucasian and Asian. Subgroup analysis was stratified by a source of control and genotype methods.

Heterogeneity assumption was evaluated with a chi-square-based *Q*-test. The statistical significance of the summary OR was determined with the *Z*-test. The heterogeneity amongst the studies was checked using the chi-square based Q statistic and considered statistically significant at *P*<0.10. When *P* for heterogeneity test (*P*_h_)>0.10, the pooled OR of each study was calculated using the fixed-effects model (the Mantel–Haenszel method, which weighs the studies by the inverse of the variance of estimates); otherwise, the random-effects model (the DerSimonian and Laird method) was used [29,30]. Funnel plot asymmetry was assessed using Begg’s test and publication bias was assessed using Egger’s test with *P*<0.05 considered as statistically significant [31]. The departure of frequencies of TNF-α gene -238 G/A polymorphism from expectation under HWE was assessed by χ^2^ test in controls using the Pearson chi-square test, again with *P*<0.05 considered as significant [32]. The power of our meta-analysis was calculated by a program named PS: Power and Sample Size Calculation (http://biostat.mc.vanderbilt.edu/wiki/Main/PowerSampleSize#Windows). All statistical tests for this meta-analysis were performed with Stata software (version 10.0; StataCorp LP, College Station, TX) [33].

### Genotyping methods

Genotyping of TNF-α gene -238 G/A was conducted using different techniques in different studies: GeneChip, Sequence, Taqman, Snapshot, PCR-restriction fragment length polymorphism (RFLP), and PCR-based denaturing HPLC (DHPLC).

### *In silico* analysis of TNF expression

To further explore the association between TNF-α expression and GC, we used the bioinformatics web: GEPIA (http://gepia.cancer-pku.cn/) [34], which provided the RNA sequencing expression data of 33 different types of tumors and corresponding healthy samples from the TCGA and the GTEx public database.

## Results

### Eligible studies

**Figure 1:**
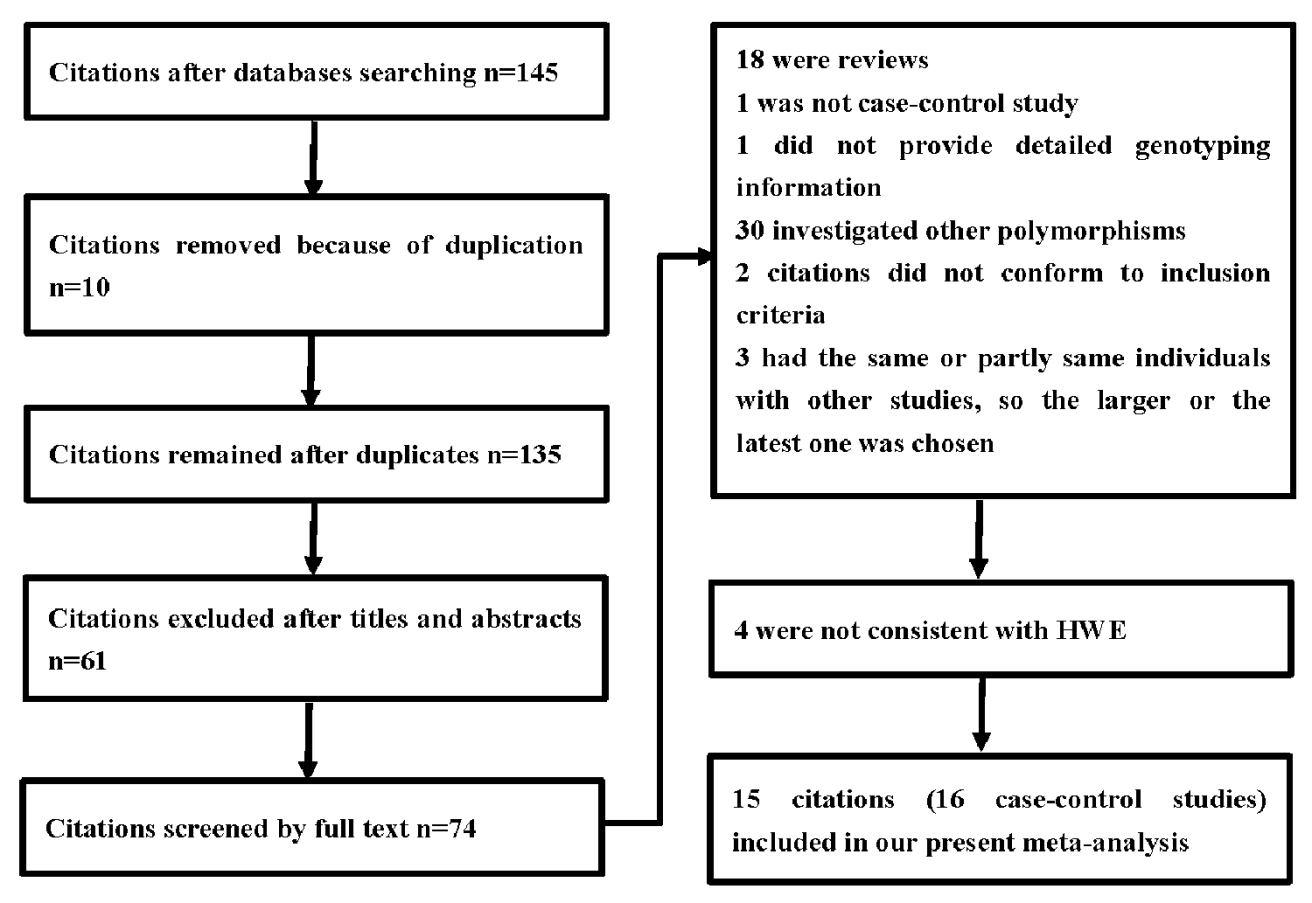
A flowchart illustrating the search strategy used to identify association studies for TNF-α gene -238 G/A polymorphism and GC risk

A total of 145 articles were retrieved based on our selection strategy from the PubMed, Embase, Web of Science, Google Scholar, WanFang, and CNKI databases. Amongst them, 10 duplicated articles were excluded; 61 articles were excluded after reviewing the title or abstract. Seventy-four articles were then downloaded for review of the full text. Amongst them, 55 articles were excluded, 1 was not a case–control study, 1 did not provide detailed genotyping information, 30 investigated other polymorphisms within the TNF gene, 2 articles did not conform to inclusion criteria, and 3 articles contained the same or partly same individuals with other studies, the larger or the latest one was chosen (Figure 1). After the above screening, 19 articles were included. Furthermore, to ensure the rigor of our study, four articles were excluded because HWE was less than 0.05 [18,19,21,22]. Thus, 15 articles about 16 case–control studies with 3309 cases and 5170 controls for TNF-α gene -238 G/A polymorphism were included in our meta-analysis [20,24,35–46] (Table 1). The 16 studies could be stratified according to the following independent criteria: (i) Source of controls: nine were HB and seven were PB; (ii) Country: seven were from China, ten were Asians, and six were Caucasians; (iii) Genotype methods: six using PCR-RFLP, two using Taqman, and three using Sequencing.

**Table 1:** Basic information for included studies of the association between *TNF-α -238G/A* polymorphism sites and GC susceptibility

Author	Year	Country	Ethnicity (1)	Ethnicity (2)	Case	Control	SOC	Case			Control			HWE	Genotype	Genotype
								AA	AG	GG	AA	AG	GG		methods (1)	methods (2)
Zeng	2006	China	China	Asian	130	142	HB	0	46	84	0	26	116	0.23	geneChip	Others
Lu	2005	China	China	Asian	250	300	PB	1	27	222	0	23	277	0.49	PCR-DHPLC	Others
Yang	2009	Korea	Not China	Asian	83	331	PB	0	10	73	0	26	305	0.46	SNaPshot	Others
Xu	2017	China	China	Asian	296	319	HB	0	31	265	0	25	294	0.47	PCR-RFLP	PCR-RFLP
Jang	2001	South Korea	Not China	Asian	52	92	HB	0	2	50	1	11	80	0.39	PCR-RFLP	PCR-RFLP
Zambon	2005	Italy	Not China	Caucasian	129	644	HB	3	13	113	1	74	569	0.38	PCR-RFLP	PCR-RFLP
Glas	2004	Germany	Not China	Caucasian	88	145	HB	0	9	79	0	11	134	0.63	PCR-RFLP	PCR-RFLP
Zang	2009	China	China	Asian	296	319	PB	0	31	265	0	25	294	0.47	PCR-RFLP	PCR-RFLP
Lee	2004	Korea	Not China	Asian	341	261	PB	0	29	312	0	25	236	0.42	PCR-RFLP	PCR-RFLP
Bai	2009	China	China	Asian	114	119	HB	3	17	94	0	9	110	0.67	Sequence	Sequence
Essadik	2015	Morocco	Not China	Caucasian	93	74	HB	0	5	88	1	15	58	0.97	Sequence	Sequence
Crusius	2008	France	Not China	Caucasian	424	1123	PB	2	27	395	5	114	1004	0.37	Sequence	Sequence
Garcia-Gonzalez	2007	Spain	Not China	Caucasian	404	404	PB	1	66	337	9	65	330	0.01	Taqman	Taqman
Hou	2007	U.S.A.	Not China	Caucasian	299	412	PB	0	24	275	0	27	385	0.49	Taqman	Taqman
Yin	2012	China	China	Asian	91	230	HB	0	7	84	0	29	201	0.31	Snapshot	Others
Yin	2012	China	China	Asian	219	255	HB	0	18	201	0	9	246	0.77	Snapshot	Others
Whiteman	2010	Australia	Not China	Caucasian	296	1299	PB	0	26	270	9	125	1165	<0.01	SpectroChip	Others
Kamangar	2006	U.S.A.	Not China	Caucasian	115	210	PB	3	6	106	2	5	203	<0.01	Taqman	Taqman
Wu	2003	Taiwan	China	Asian	220	230	HB	3	4	213	4	2	224	<0.01	Sequence	Sequence
Li	2012	China	China	Asian	300	300	HB	25	11	264	12	14	274	<0.01	MALDI-TOF-MS	Others
Xing	2006	China	China	Asian	130	142	HB	0	46	84	0	26	116	0.23	geneChip	Others
Wu	2002	Taiwan	China	Asian	150	220	HB	2	4	144	2	4	214	<0.01	Sequence	Sequence
Wu	2004	Taiwan	China	Asian	204	210	HB	2	3	199	2	3	205	<0.01	Sequence	Sequence

Abbreviations: PCR-DHPLC, PCR-based DHPLC; PCR-RFLP, PCR and restrictive fragment length polymorphism.

**Figure 2:**
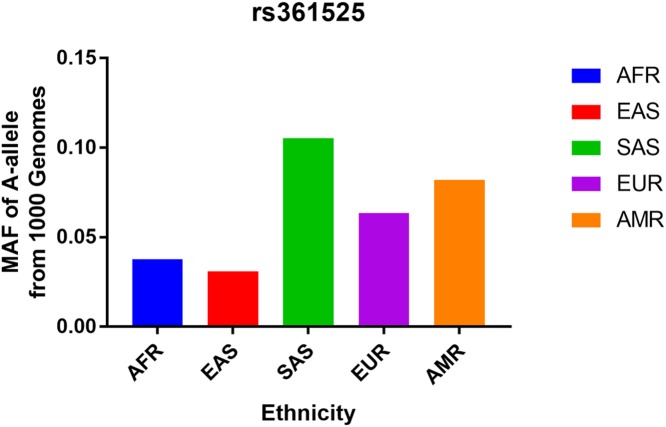
The MAF of minor-allele (mutant-allele) for TNF-α gene -238 G/A polymorphism from the 1000 Genomes online database and present analysis Abbreviations: AFR, African; AMR, American; EAS, East Asian; EUR, European; SAS, South Asian.

We also checked the minor-allele frequency (MAF), the frequency of the most common mutant allele, of the TNF-α gene -238 G/A polymorphism amongst the five main population groups reported in the 1000 Genomes Browser: East Asian (0.0308), European (0.0636), African (0.0378), American (0.0821), and South Asian (0.1053) (Figure 2). The MAFs in our analysis were found to be 0.05772 and 0.05299 for the case and control groups, respectively. Our estimates accorded well with the East Asian MAF reported in the 1000 Genomes Browser database. Finally, the distribution of genotypes amongst controls was consistent with HWE in all models.

### Meta-analysis

In the pooled analysis of all populations, no association could be observed between GC risk and the genotypic variants of TNF-α gene -238 G/A polymorphism. The different genetic models of polymorphism-associated risk tested for the whole population were: allelic contrast (OR = 1.13, 95% CI = 0.86–1.47, *P*_heterogeneity_<0.01, *P*=0.384, Figure 1), heterozygote comparison (OR = 1.11, 95% CI = 0.85–1.45, *P*_heterogeneity_<0.01, *P*=0.423), and the dominant allele model (OR = 1.13, 95% CI = 0.86–1.48, *P*_heterogeneity_<0.01, *P*=0.396) (Table 2).

**Table 2:** Total and stratified subgroup analysis for *TNF-α -238G/A* polymorphism sites and GC susceptibility

Variables	*n*	Case/ control	A-allele compared with G-allele			AG compared with GG			AA+AG compared with GG		
			OR (95% CI)	*P*_h_	*P*	OR (95% CI)	*P*_h_	*P*	OR (95% CI)	*P*_h_	*P*
Total	16	3309/5170	1.13 (0.86–1.47)	0.000	0.384	1.11 (0.85–1.45)	0.000 0.423		1.13 (0.86–1.48)	0.000	0.396
Ethnicity											
Asian	10	1872/2368	**1.39 (1.02–1.89)**	**0.024**	**0.039**	**1.38 (1.02–1.88)**	**0.041**	**0.040**	**1.40 (1.02–1.93)**	**0.024**	**0.040**
Caucasian	6	1437/2802	0.84 (0.58–1.23)	0.016	0.372	0.82 (0.56–1.20)	0.033	0.317	0.83 (0.57–1.21)	0.028	0.330
China	7	1396/1684	**1.59 (1.27–2.00)**	**0.116**	**0.000**	**1.57 (1.24–1.99)**	**0.133**	**0.000**	**1.61 (1.27–2.03)**	**0.104**	**0.000**
Not China	9	1913/3486	0.86 (0.63–1.18)	0.015	0.359	0.86 (0.63–1.17)	0.035	0.332	0.86 (0.63–1.17)	0.026	0.334
Source of Control											
HB	9	1212/2020	1.15 (0.71–1.85)	0.000	0.566	1.10 (0.67–1.80)	0.000	0.712	1.13 (0.68–1.86)	0.000	0.634
PB	7	2097/3150	1.03 (0.79–1.34)	0.072	0.854	1.00 (0.83–1.21)	0.111	0.975	1.04 (0.80–1.34)	0.092	0.774
Genotype methods											
PCR-RFLP	6	1202/1780	1.14 (0.88–1.46)	0.339	0.321	1.07 (0.83–1.40)	0.364	0.591	1.11 (0.85–1.44)	0.371	0.445
Others	5	773/1258	**1.60 (1.20–2.12)**	**0.114**	**0.001**	**1.58 (1.00–2.52)**	**0.072**	**0.051**	**1.60 (1.01–2.53)**	**0.073**	**0.045**
Taqman	2	703/816	0.90 (0.67–1.20)	0.197	0.468	1.06 (0.78–1.45)	0.520	0.701	0.98 (0.72–1.33)	0.326	0.882
Sequence	3	631/1316	0.76 (0.22–2.55)	0.000	0.652	0.68 (0.23–2.02)	0.003	0.492	0.71 (0.22–2.34)	0.001	0.577

Abbreviations: *P*_h_, value of *Q*-test for heterogeneity test; *P, Z*-test for the statistical significance of the OR. The bold values represent the significance of association between TNF-α -238G/A polymorphism sites and GC susceptibility.

**Table 3:** Publication bias tests (Begg’s funnel plot and Egger’s test for publication bias test) for *TNF-α -238G/A* polymorphism

Egger’s test						Begg’s test	
Genetic type	Coefficient	S.E.M.	*t*	*P*-value	95% CI of intercept	*z*	*P*-value
A-allele compared with G-allele	0.300	1.379	0.22	0.831	(−2.659, 3.259)	0.18	0.857
AG compared with GG	0.156	1.385	0.11	0.912	(−2.814, 3.126)	−0.05	0.964
AA+AG compared with GG	−0.214	1.489	0.14	0.888	(−3.031, 3.461)	−0.05	1

**Figure 3:**
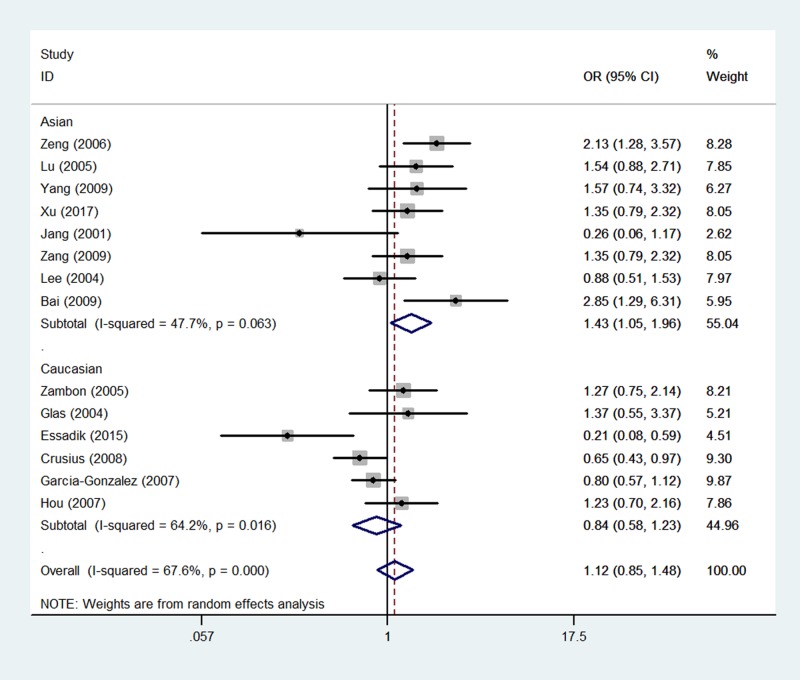
Forest plot of GC risk associated with TNF-α gene -238 G/A polymorphism (A-allele compared with G-allele) in the ethnicity subgroup The squares and horizontal lines correspond to the study-specific OR and 95% CI. The area of the squares reflects the weight (inverse of the variance). The diamond represents the summary OR and 95% CI.

**Figure 4:**
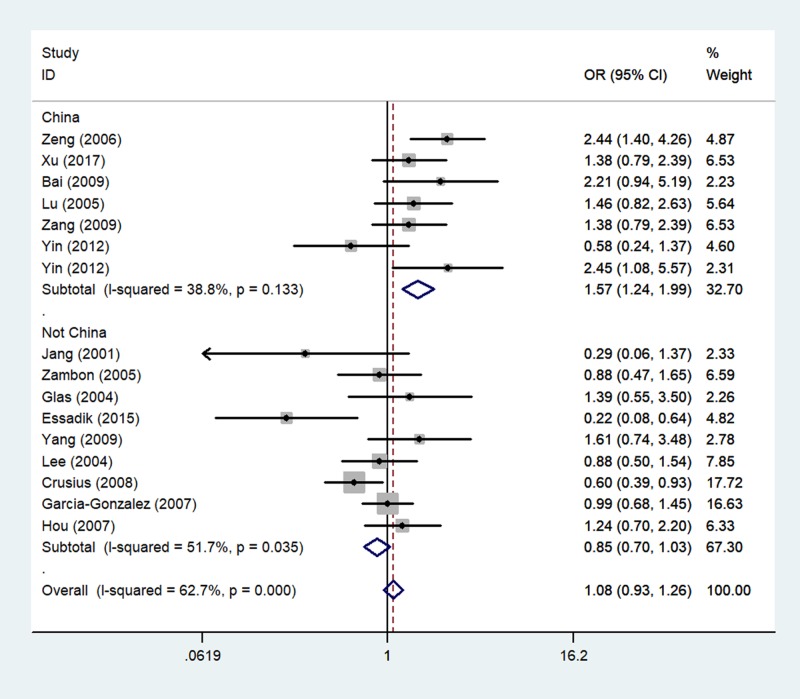
Forest plot of GC risk associated with TNF-α gene -238 G/A polymorphism (AG compared with GG) by country type The squares and horizontal lines correspond to the study-specific OR and 95% CI. The area of the squares reflects the weight (inverse of the variance). The diamond represents the summary OR and 95% CI.

In the analysis stratified by ethnicity, significantly increased associations were found between TNF-α gene -238 G/A polymorphism and GC risk in the Asian ethnicity (A-allele compared with G-allele: OR = 1.39, 95% CI = 1.02–1.89, *P*_heterogeneity_=0.024, *P*=0.039, Figure 3; AG compared with GG: OR = 1.38, 95% CI = 1.02–1.88, *P*_heterogeneity_=0.041, *P*=0.040; AA+AG compared with GG: OR = 1.40, 95% CI = 1.02–1.93, *P*_heterogeneity_=0.024, *P*=0.040). In the analysis stratified by country, similarly significant associations were detected between TNF-α gene 308 G/A polymorphism and GC risk in the Chinese population (A-allele comapred with G-allele: OR = 1.59, 95% CI = 1.27–2.00, *P*_heterogeneity_=0.116, *P*=0.000; AG compared with GG: OR = 1.57, 95% CI = 1.24–1.99, *P*_heterogeneity_=0.133, *P*=0.000, Figure 4; AA+AG compared with GG: OR = 1.61, 95% CI = 1.27–2.03, *P*_heterogeneity_=0.104, *P*=0.000) (Table 2).

**Figure 5:**
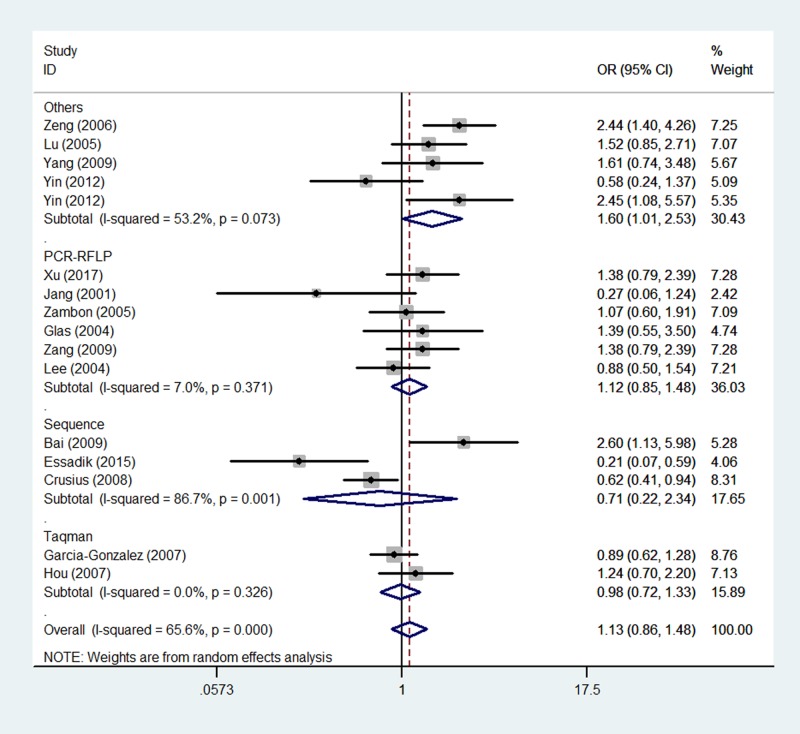
Forest plot of GC risk associated with TNF-α gene -238 G/A polymorphism (AA+AG compared with GG) by sgenotype methods The squares and horizontal lines correspond to the study-specific OR and 95% CI. The area of the squares reflects the weight (inverse of the variance). The diamond represents the summary OR and 95% CI. Each point represents a separate study for the indicated association. Log [OR], natural logarithm of OR. Horizontal line, mean effect size.

In the analysis stratified by genotype methods subgroup, a similar magnitude of association was observed between TNF-α gene -238 G/A polymorphism and GC risk in the group for others (A-allele compared with G-allele: OR = 1.60, 95% CI = 1.20–2.12, *P*_heterogeneity_=0.114, *P*=0.001; AG compared with GG: OR = 1.58, 95% CI = 1.00–2.52, *P*_heterogeneity_=0.072, *P*=0.051; AA+AG compared with GG: OR = 1.60, 95% CI = 1.01–2.53, *P*_heterogeneity_=0.073, *P*=0.045, Figure 5). However, no significant associations were found for PCR-RFLP and Taqman methods (Table 2).

### Sensitivity analysis and publication bias

**Figure 6:**
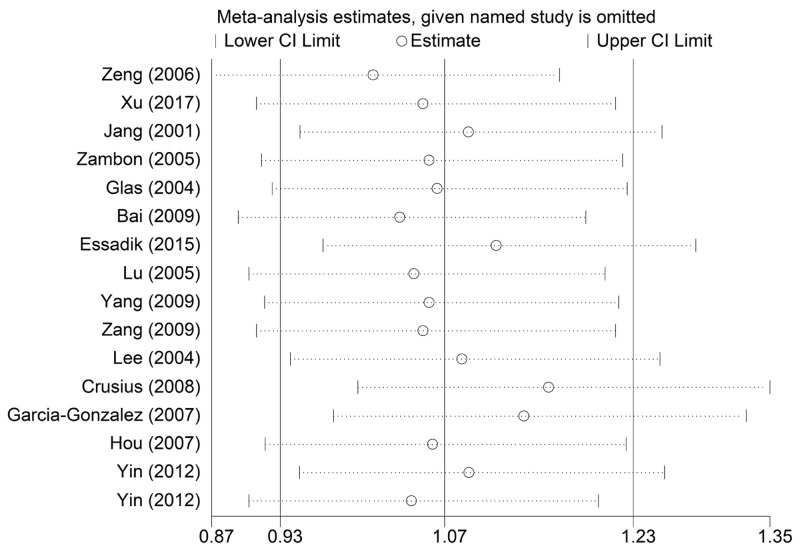
Sensitivity analysis between TNF-α gene -238 G/A polymorphism and GC risk (A-allele compared with G-allele)

**Figure 7:**
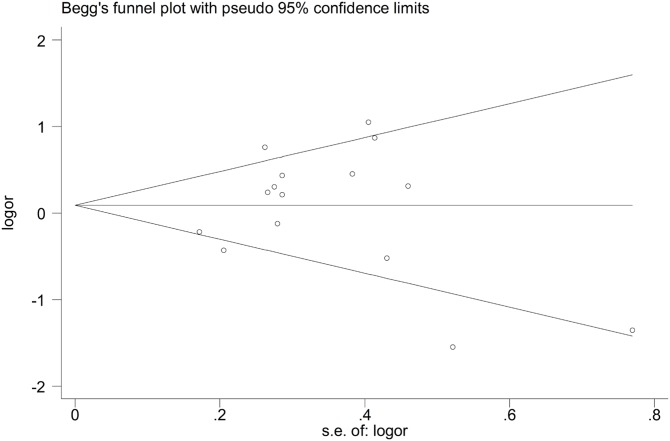
Begg’s funnel plot for publication bias test (A-allele compared with G-allele)

**Figure 8:**
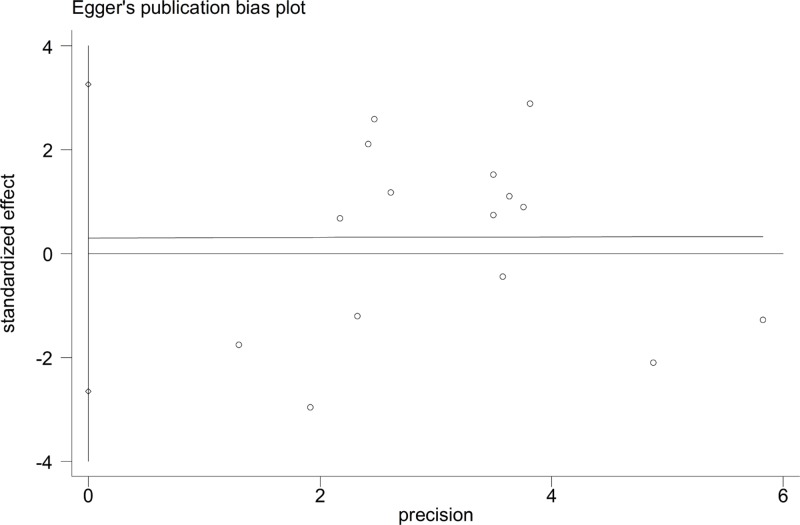
Egger’s publication bias plot (A-allele compared with G-allele)

Sensitivity analysis was performed to assess the influence of each individual study on the pooled OR by sequential removal of individual studies. The results suggested that no individual study affected the overall OR significantly (Figure 6). This suggests that our conclusion is credible and generalizable. Begg’s funnel plot and Egger’s test were performed to assess publication bias. As shown in Table 3, the shapes of the funnel plots did not reveal an obvious asymmetry in any of the comparison models. Similarly, neither of the above tests provided any evidence of publication bias (A-allele compared with G-allele: *t* = 0.22, *P*=0.831 for Egger’s test; and *z* = 0.18, *P*=0.857 for Begg’s test, Figure 7,8; AG compared with GG: *t* = 0.11, *P*=0.912 for Egger’s test; and *z* = −0.05, *P*=0.964 for Begg’s test; AA+AG compared with GG: *t* = 0.14, *P*=0.888 for Egger’s test; and *z* = −0.05, *P*=1 for Begg’s test).

### *In silico* analysis of TNF expression

**Figure 9:**
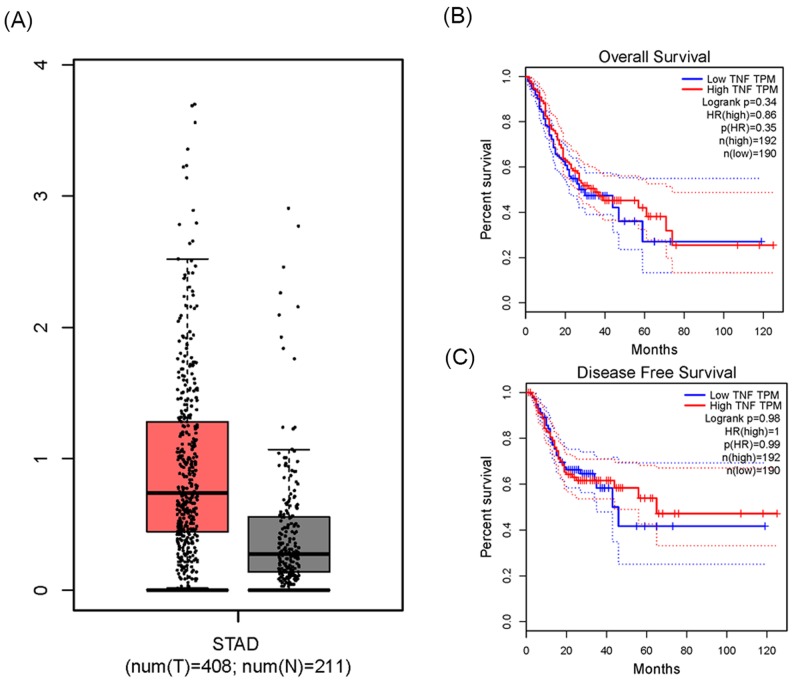
The expression and prognostic analysis of TNF gene about gastric cancer from bioinformatics *In silico* analysis of TNF expression: (**A**) the relative expression of TNF in GC tissue and normal tissue using TCGA database. (**B**) The correlation between TNF expression levels and overall survival time of GC patients. TPM stands for the expression of TNF in each tissue. *P*-value less than 0.05 was considered as statistically significant. (**C**) The correlation between TNF expression levels and disease-free survival time of GC patients.

*In silico* results indicated that the expression of TNF in GC tissue was higher than in normal stomach (TPM: transcripts per kilobase million = 0.76 compared with 0.23 respectively, *P*<0.01, Figure 9A). In addition, we analyzed whether TNF expression affects the overall survival time and disease-free survival time of GC patients. The Kaplan–Meier estimate showed no significant difference in either the overall survival time (Log-rank *P*=0.34, *p*HR = 0.35, Figure 9B) or the disease-free survival time between the low and high TNF TPM groups (Log-rank *P*=0.98, *p*HR = 0.99, Figure 9C).

## Discussion

GC remains a high-incidence malignance and the incidence greatly varies between countries. The majority of cases are registered in developing countries, with half of them reported in Eastern Asia. In addition, GC incidence is twice as high in men as in women. This suggests that race, environmental, hormonal, or genetic factors may affect GC risk [47]. Furthermore, to the best of our knowledge, host genetic factors are emerging as key determinants of disease for many cancers. Polymorphisms in pro-inflammatory cytokine genes, especially TNF-α and its receptor, are associated with an increased risk of GC [1,9,35]. Therefore, we selected a controversial SNP named TNF-α gene -238 G/A polymorphism to analyze the susceptibility of individuals to GC.

Four previous meta-analyses [14–16,48] focussed on this polymorphism, however, some fundamental flaws existed within these studies. For example a meta-analysis from Zheng et al. [48], compared with the previous meta, some improvements were shown in our analysis: first, several studies were not included in their study (Xu et al. (2017), Bai et al. (2009), Essudik et al. (2015), Hou et al. (2007)); second, seven case–control studies were not consistent with HWE (Wu et al. (2002, 2003, 2004), Kamangar et al. (2006), Whiteman et al. (2010), Li et al. (2012), Xing et al. (2006)); third, Wu et al. (2002, 2003, 2004) had some duplicated data, which may improve the powerful and credible. Above two changes were the degrees of innovation and creation. So thus a comprehensive analysis should be carried out. In the present study, 16 strictly case–control studies [20,24,35–46], including 3309 cases and 5170 controls about the association between TNF-α gene -238 G/A polymorphism and GC risk, were calculated. Finally, we found that A-allele may act as a risk factor in Asians, especially Chinese. To our knowledge, GC in East Asian countries such as China still poses a major threat to the health of patients across the world. Of note, the majority of worldwide GC cases and deaths annually occur in China, which accounts for a stunning 42.5% of worldwide cases and 45.0% of worldwide deaths [49,50]. To combine our results, it is logical to highlight this polymorphism, which may become an early diagnosis and biomarker-based target for GC.

In our *in silico* analysis, the expression of TNF-α was higher in GC tissues, which was similar to carcinogenic genes and was consistent with previous publications. Maolake et al. [51] suggested TNF-α might increase the metastatic potential of prostate cancer cells in lymph node metastasis through CCL2/CCR7 axis. Ma et al. [52] showed that TNF-α levels were correlated with clinical disease stage and lymph node metastasis in breast cancer patients. At last, Sahibzada et al. [53] reported that the excessive expression of TNF-α plays a role in oral cancer progression and establishment of angiogenesis. In addition, the -238A allele of TNF caused a significant increase in transcription following a transient expression assay in mitogen-stimulated Jurkat and Raji cells [54], indicating that the -238A allele and/or other TNF-α gene polymorphism sites may increase the expression of TNF-α. These previous reports highlight that TNF-α gene polymorphisms, especially -238A allele might act as a risk factor for cancer development and progression, which was consistent with our conclusions.

Meta-analyses have been recognized as an effective method to summarize and review previously published quantitative research to answer a wide variety of clinical questions [55]. However, several limitations in our meta-analysis should be acknowledged. First, the sample of published studies remains small for a comprehensive analysis. Second, the source of articles is uneven in geographical distribution, which may introduce ethnicity bias, and further studies should focus on Africans and Caucasians. Third, none of the articles provide information related to TNF expression levels classified by gender or stage of the tumor and therefore, we could not analyze these factors. We advocate for future articles including this information.

In summary, our meta-analysis showed that TNF-α gene -238 G/A polymorphism was associated with significantly increased GC risk in East Asian populations, especially Chinese. Furthermore, well-designed and larger studies, dealing specifically with gene–gene and gene–environment interactions, are warranted.

## Abbreviations

CI: confidence interval
GC: gastric cancer
HB: hospital-based
HWE: Hardy–Weinberg equilibrium
MAF: minor-allele frequency
PB: population-based
RFLP: restriction fragment length polymorphism
SNP: single-nucleotide polymorphism
TNF-α: tumor necrosis factor α
TPM: transcript per kilobase million
